# Chinese Medicine and Surgery in New York

**Published:** 1904-10

**Authors:** Nelson W. Wilson

**Affiliations:** Buffalo, N. Y


### Chinese Medicine and Surgery in New York.

By NELSON W. WILSON, M. D., Buffalo, N. Y.

DURING several years' work among the Chinese in New York, years during which a daily and intimate relationship with the children of the Sun amounted to almost a residence, the writer had opportunity to gather a smattering of the Chinese methods of administering drugs and treating disease. Just as Mrs. Bishop says in her very interesting résumé of practice in the Flowery Kingdom, the Chinese doctor makes his diagnosis on the pulses "above and below the wrist" and it was a novel experience to see a native physician spend something like an hour on the twelve pulses of a patient who was ill—very ill from the effects of a combination of Chinese opium and American beer.

In those halcyon days when the pungent odor of the pipe and the aroma of the spices and the herbs mingled with the frou-frou of the skirts and the shuffle of sandaled feet, the Chinese sick were doctored by a little undersized medicine man who lived in Chinese style and wore a queue but garbed himself in American clothing, Dr. Charley Sing. His shop was a dark room on Mott street, next to the King restaurant, which in later years became famous as the one last resort of fashionable slummers who spent foolish sums of money for cheap chop seuy and deer tendon and funny little Chinese potatoes and the common every day lichee nuts and went back and gossiped with their less fortunate sisters and cousins and aunts of the terrible things they had eaten in the way of heathen cooking.

Charley Sing knew Chinese medicine evidently for his drugs were the most outlandish looking affairs one could imagine. There were in the mixtures he put up dried toads and lizards and powdered horn of various animals and spiders and snake meat and numerous herbs. The crudity of Chinese practice is best illustrated by the fact that all the medicines and ingredients he used were in the natural state when received from China and he prepared all the mixtures he administered. There were little glass and earthern jars, containing whole spiders and whole toads and lizards, dead and dried, but retaining sufficient of their original form to be easily recognisable. In the case referred to above an emetic was used. It was an herb, mixed with a pint of hot water which the patient drank and as promptly expelled.

For rheumatism there is a black, pasty mixture which comes in little earthern jars holding less than an ounce. The dose is relatively half a teaspoonful; sometimes in severe cases it is a teaspoonful or a teaspoon and a half. This is administered internally and the patient is wrapped up tightly in sheets and blankets and watched. He perspires freely; not the usual watery perspiration, but a thick, almost mucilaginous exudate, which is brownish in color and terribly odoriferous. What the drug is could not be learned with any degree of clearness but the idea was conveyed that it was the gum of a tree. There is danger of an overdose producing heart failure, hence the patient is closely watched. When the exudate is profuse, the patient is bathed in hot water and wrapped up again and left to recover from the effects of the drug, which leaves him in a very much weakened state. His recovery is usually rapid and it is rare, the Chinese claim, that a second treatment is necessary. The case of an American suffering from rheumatism came under the writer's observation. The white man was cured with half a teaspoonful dose. That was quite ten years ago and there has been no recurrence of the disease. With respect to ignorance and nauseous dosage the Chinese physician and the Indian medicine man are brothers.

As regards surgery the Chinese of recent years have become rather more enlightened. They now do minor surgery, but it will probably be years before they grow to major operations. In battle, the wounded are left to care for themselves and reports from the interior credit the well with killing the desperately wounded.

The fatalism and fanaticism of the Chinese makes this almost a blithesome task. It relieves an active and armed body of the impedimenta of wounded. One rarely sees a cripple among the Chinese. Practising physicians devote most of their surgical skill to acupuncture which is really the national surgery. It is used for everything—fractures, cholera, smallpox and even sore eyes. The surgical education of the student consists of the study of a bronze manikin full of tiny holes corresponding to the proper points of puncture. When a student is examined he is placed before this figure which has been covered with paper and his examiner names a disease which he is supposed to treat. To pass the examination the student must plunge the needle into the proper hole in the manikin.

The only surgical operation the writer witnessed in Chinatown was the opening of an abscess. A long needle with an incomplete ring at the end and a small, sharp pointed knife, much like our own straight bistoury, were the instruments used. The ring of the needle was placed over the abscess and pressed down until a little ball of tissue projected, through which the point of the knife was passed into the abscess cavity. The opening was then smeared over with a dirty looking paste.

The only true Chinese major operation known was recently described in *American Medicine.* This cannot be regarded as a "surgical" procedure. The Imperial Court has had attached to it for centuries an army of self-constituted martyrs numbering in the neighborhood of 3,000. These are eunuchs. In addition, the princes and princesses are entitled to 30 each; nephews and young children of the reigning Emperor to 20 each and the vast army of cousins to 10 each. The direct descendents of the eight Manchoo princes who aided in the establishment of the present dynasty are entitled to 10 each.

The privileges of the position of eunuch are so well established that boys are sold by their parents and young married men offer themselves for the operation of emasculation. An anesthetic is unheard of and the operation is bereft of any suspicion of cleanliness.

The prospective eunuch is bound to a narrow cot; his shoulders are grasped and held by an assistant while his legs are held apart by two others. The operator grasps the penis and scrotum at the root in his left hand compressing and twisting them and expelling as much blood as possible. When ready he asks of the parents—in the case of a young boy: "Do you consent?" If the answer is in the affirmative he removes with a single sweep of his knife, the penis and scrotum with, of course, the contents of the latter, and as close to the pubo-perineal surface as possible. Sometimes a small hatchet is used; but as a rule the instrument is a knife, straight or sickle-shaped.

In spite of the revolting cruelty of the operation and its horrifying uncleanliness, the mortality is reported to be not more than 3 or 4 per cent. There is naturally a tendency to cicatricial atresia of the urethral orifice and this oftentimes proves troublesome, especially in the younger subjects. The most frequent complication is incontinence of urine. This is tolerated by the operator for a reasonable length of time; but if it persists beyond a certain date the patient is beaten everytime the accident occurs and he is rapidly cured. For the prevention of cicatricial contraction of the urethra a wooden and sometimes a metal bougie is used. The organs removed during the operation are preserved with religious care and are buried with the owner, to prevent his soul being transformed into a mule, which is the fate of all eunuchs who arrive at the end of the Great Journey without their sexual organs.

When the wound has healed there is a triangular scar with its apex below. Often the final results of the operation are partial cicatricial stricture of the urethra with partial incontinence of urine, followed by vesical catarrh and frequently the formation of a vesical calculus. The odor resulting from this partial incontinence of ammoniacal urine is so well-known in Pekin that among the French residents the saying: "Il pue comme un eunuque; on le sent à cinq cents pas" is a popular one.

Before wholly damning Chinese medicine and dismissing it one must remember that the Chinese claim that for over four thousand years mercury has been used for the cure of syphilis.

49 NIAGARA STREET.

---

### NERVOUS EXCITEMENT AND NEURALGIA.

℞ Zinc valerianate .................... gr. 5-6
Extract of hyoscyamus .................... gr. 1-3
Extract of belladonna .................... gr. 1-6

For one pill. Three pills daily.

Or:

℞ Monobromated camphor
Quinine valerianate, a a .................... gr. iss
Extract of hyoscyamus .................... gr. 1-3
Extract of belladonna .................... gr. 1-6

For one pill. Four or five pills daily.

—*Herzen in Medical Bulletin; Southern Medicine and Surgery.*

